# *Bacillus velezensis:* A Valuable Member of Bioactive Molecules within Plant Microbiomes

**DOI:** 10.3390/molecules24061046

**Published:** 2019-03-16

**Authors:** Muhammad Fazle Rabbee, Md. Sarafat Ali, Jinhee Choi, Buyng Su Hwang, Sang Chul Jeong, Kwang-hyun Baek

**Affiliations:** 1 Department of Biotechnology, Yeungnam University, Gyeongsan 38541, Gyeongbuk, Korea; rabbi.biotech@gmail.com (M.F.R.); sarafatbiotech@ynu.ac.kr (M.S.A.); woolde@naver.com (J.C.); 2 Nakdonggang National Institute of Biological Resources, Sangju 37242, Gyeongbuk, Korea; hwang1531@nnibr.re.kr (B.S.H.); j1685@nnibr.re.kr (S.C.J.)

**Keywords:** *Bacillus velezensis*, bioactive compound, volatile organic compound, induced systemic resistance

## Abstract

*Bacillus velezensis* is an aerobic, gram-positive, endospore-forming bacterium that promotes plant growth. Numerous strains of this species have been reported to suppress the growth of microbial pathogens, including bacteria, fungi, and nematodes. Based on recent phylogenetic analysis, several *Bacillus* species have been reclassified as *B. velezensis*. However, this information has yet to be integrated into a well-organized resource. Genomic analysis has revealed that *B. velezensis* possesses strain-specific clusters of genes related to the biosynthesis of secondary metabolites, which play significant roles in both pathogen suppression and plant growth promotion. More specifically, *B. velezensis* exhibits a high genetic capacity for synthesizing cyclic lipopeptides (i.e., surfactin, bacillomycin-D, fengycin, and bacillibactin) and polyketides (i.e., macrolactin, bacillaene, and difficidin). Secondary metabolites produced by *B. velezensis* can also trigger induced systemic resistance in plants, a process by which plants defend themselves against recurrent attacks by virulent microorganisms. This is the first study to integrate previously published information about the *Bacillus* species, newly reclassified as *B. velezensis*, and their beneficial metabolites (i.e., siderophore, bacteriocins, and volatile organic compounds).

## 1. Introduction

Rhizosphere is a highly competitive environment, where microorganisms constantly battle for resources to survive [1]. The term rhizosphere was first coined by Lorentz Hiltner in 1904 [2] to describe the nutrient-rich zone of soil (usually 1–3 mm around roots), where plant root exudates facilitate colonization by microbial communities [1,3]. Some such rhizosphere-associated bacteria, i.e., plant growth-promoting rhizobacteria (PGPR), are recognized for their ability to promote plant weight and crop yield [4], by (1) producing antimicrobial secondary metabolites (i.e., antagonism), (2) competing for niches and nutrients (i.e., colonization), and (3) stimulating induced systemic resistance (ISR) [5].

*Bacillus* species are considered important PGPR, producing a vast array of biologically active secondary metabolites that can potentially inhibit the growth of plant pathogens and deleterious rhizospheric microorganisms [5]. *Bacillus* spp. are preferred in agricultural systems, due to their ability to form endospores, which can survive to heat exposure and desiccation, and capacity to be formulated into stable dry powders with long shelf lives [6]. Furthermore, because *Bacillus* spp. are already common inhabitants of plant root microflora, *Bacillus* spore-based biocontrol agents have little, if any, effect on the composition of the plant root microbial communities [7]. Several *Bacillus*-based products are now commercially available, including RhizoVital^®^ (*Bacillus amyloliquefaciens* FZB42; ABiTEP, GmbH, Berlin, Germany), Amylo-X^®^ WG (*B. amyloliquefaciens* subsp. *plantarum* D747; Certis Europe BV, Netherlands), RhizoPlus^®^ (*B. subtilis* FZB24; ABiTEP), Sonata^®^ (*B. pumilus* QST2808; AgraQuest, Inc., Davis, California, USA), Taegro^®^ (*B. subtilis* var. *amyloliquefaciens* FZB24; Novozymes Biologicals, Inc., Salem, Virginia, USA [8].

Recently, different strains of *B. velezensis,* which is a typical PGPR, have received considerable attention. For example, living spores of *B. amyloliquefaciens* FZB42, now reclassified as a strain of *B. velezensis*, have been formulated into the commercially available bio-inoculant RhizoVital^®^, which is used to control a variety of soil-borne diseases [9]. The strain is capable of stimulating plant growth and producing different types of biologically active secondary metabolites that suppress plant pathogenic microflora [10]. Meanwhile, *B. velezensis* has been formulated into the commercially available fungicide Botrybel (Agricaldes, Spain), owing to its activity against *Botrytis cinerea*, the etiological agent of gray mold, which reportedly infects over 200 plant species worldwide [11].

## 2. Emergence of *B. velezensis* from the *B. subtilis* Species Complex

**Figure 1:**
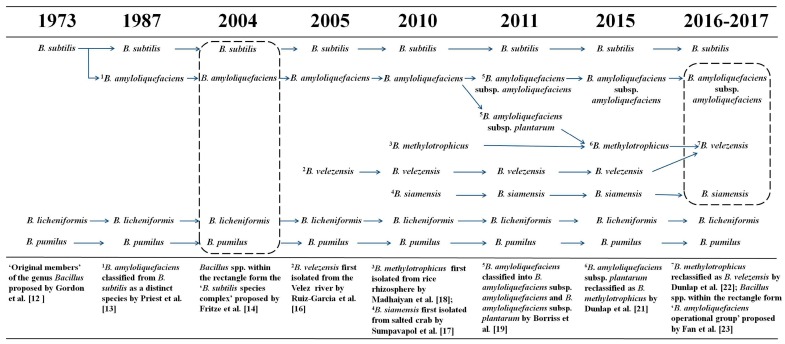
*Bacillus velezensis* is the conspecific species integrating *B. amyloliquefaciens* subsp. *plantarum* and *B. methylotrophicus* (adapted by Dunlap et al. [22]). The significance of the numbers are explained at the bottom of the same column.

*Bacillus* is a large and heterogeneous collection of aerobic or facultatively anaerobic, rod-shaped, endospore-forming bacteria that are widely distributed throughout the environment. *B. subtilis*, *B. licheniformis*, and *B. pumilus* have been identified as the ‘original members’ of the genus *Bacillus* [12] (Figure 1). In 1943, Fukumoto first isolated *B. amyloliquefaciens*, a bacterium that produces liquefying amylase from soil [13]. Later, *B. amyloliquefaciens* was combined with the closely related *B. licheniformis*, *B. pumilus*, and *B. subtilis* into the ‘*B. subtilis* species complex’, based on phylogenetic and phenetic evidence [14]. This classification was done based on the highly conserved nature of the protein-encoding sequences in *B. subtilis* species complex [15]. For many years, these closely related species were difficult to classify using classic taxonomical parameters such as morphology, physiological characteristics, guanine-cytosine content, and phylogenetic analysis through 16S rRNA gene sequencing.

*B. velezensis* (strain CR-502^T^ and strain CR-14b) was first isolated from environmental samples taken from the mouth of the Vélez River at Torredelmar in the province of Málaga, Spain [16]. Phenotypic tests and phylogenetic analyses indicated that the strains were members of the genus *Bacillus* and closely related to *B. subtilis* and *B. amyloliquefaciens*. Further DNA–DNA hybridization experiments revealed that the novel strains possessed less than 20% similarity with other *Bacillus* species and, therefore, represented a distinct species of *Bacillus* [16] (Figure 1).

Meanwhile, *B. siamensis* (strain PD-A10^T^) was first isolated from the salted crab (*poo-khem*) in Thailand [17]. As found in *B. velezensis*, phenotypic and chemotaxonomic analyses indicated that the novel strain was a member of the genus *Bacillus* (Figure 1), and the 16S rRNA gene sequence of the strain PD-A10^T^ was similar to those of both *B. amyloliquefaciens* NBRC 15535^T^ (99.5%) and *B. subtilis* DSM10^T^ (99.4%) [17].

*B. methylotrophicus* (strain KACC 13105^T^) was isolated from rice rhizospheric soil in Korea [18]. The strain was capable of utilizing methanol, trimethylamine, and ethanol as carbon sources, and was closely related to members of the *B. subtilis* species complex, with 16S rRNA gene sequence similarity values ranging from 98.2 to 99.2% [18].

In 2011, *B. amyloliquefaciens* strains were divided among the subspecies *B. amyloliquefaciens* subsp. *amyloliquefaciens* and *B. amyloliquefaciens* subsp. *plantarum*, based on complete genome analysis [19]. Strains of *B. amyloliquefaciens* subsp. *plantarum* are plant-associated and typically used as biological control agents and/or plant growth promoters [20]. Furthermore, comparative genomic analysis of *B. amyloliquefaciens* subsp. *plantarum* and *B. methylotrophicus* indicated that the genomes were highly similar (95%), with only minor differences within their genomic sequences [21]. Therefore, *B. amyloliquefaciens* subsp. *plantarum* was synonymized with *B. methylotrophicus* [21] (Figure 1), and successively, *B. methylotrophicus* was synonymized with *B*. *velezensis*, owing to the high phenotypic and genotypic coherence of the taxa [22].

The taxonomic statuses of 66 closely related *B. amyloliquefaciens* strains were assessed by comparing complete RNA polymerase beta-subunit (*rpoB*) sequences and core genome sequences [23]. The strains were clustered into a single clade, i.e., the “*B. amyloliquefaciens* operational group”, which contains three tightly linked branches: (1) *B. amyloliquefaciens* subsp. *amyloliquefaciens*, (2) *B. siamensis*, and (3) *B. velezensis*, a taxon that includes all the strains previously classified as *B. velezensis*, *B. methylotrophicus*, and *B. amyloliquefaciens* subsp. *plantarum* (Figure 1).

**Figure 2:**
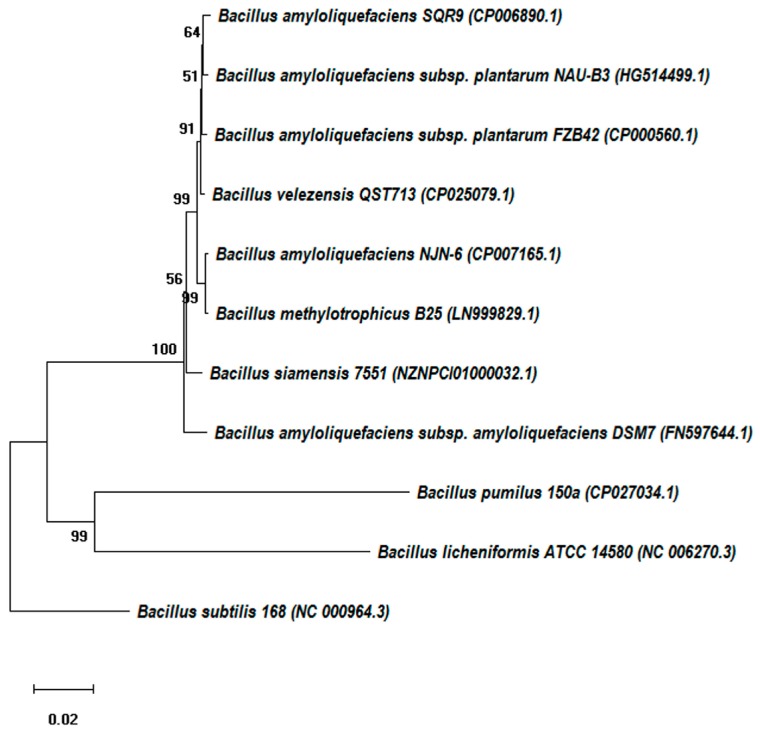
Phylogenetic tree constructed from the *rpoB* gene sequences of type strains of species from the “*B. subtilis* species complex” by the neighbor-joining method (using MEGA software). Bootstrap values (%) are given at the nodes obtained by repeating the analysis 1000 times. The scale bar indicates 0.02 nucleotide substitutions per nucleotide position.

This review focuses on *B. velezensis,* which includes a variety of previously reported strains, namely, *B. amyloliquefaciens* subsp. *plantarum* FZB42 [22], *B. amyloliquefaciens* FR203A [24], *B. amyloliquefaciens* SQR9 [25], *B. amyloliquefaciens* NJN-6 [26], *B. amyloliquefaciens* SQRT3 [27], *B. methylotrophicus* KACC 13105^T^ [22], *B. velezensis* CR-502^T^ and CR-14b [16], and *B. subtilis* GB03 [28]. Based on *rpoB* gene analysis and other analyses, these taxa are synonymous with *B. velezensis* and are each capable of suppressing pathogens. A phylogenetic tree inferred from the type strains of species from the “*B. subtilis* species complex” is presented in Figure 2. According to phylogenomic analysis, *Bacillus* species synonymous with *B. velezensis* were clustered into clades consisting of *B. amyloliquefaciens, B. amyloliquefaciens* subsp. *plantarum*, and *B. methylotrophicus.*

## 3. Bioactive Molecules Synthesized by *B. velezensis*

**Figure 3:**
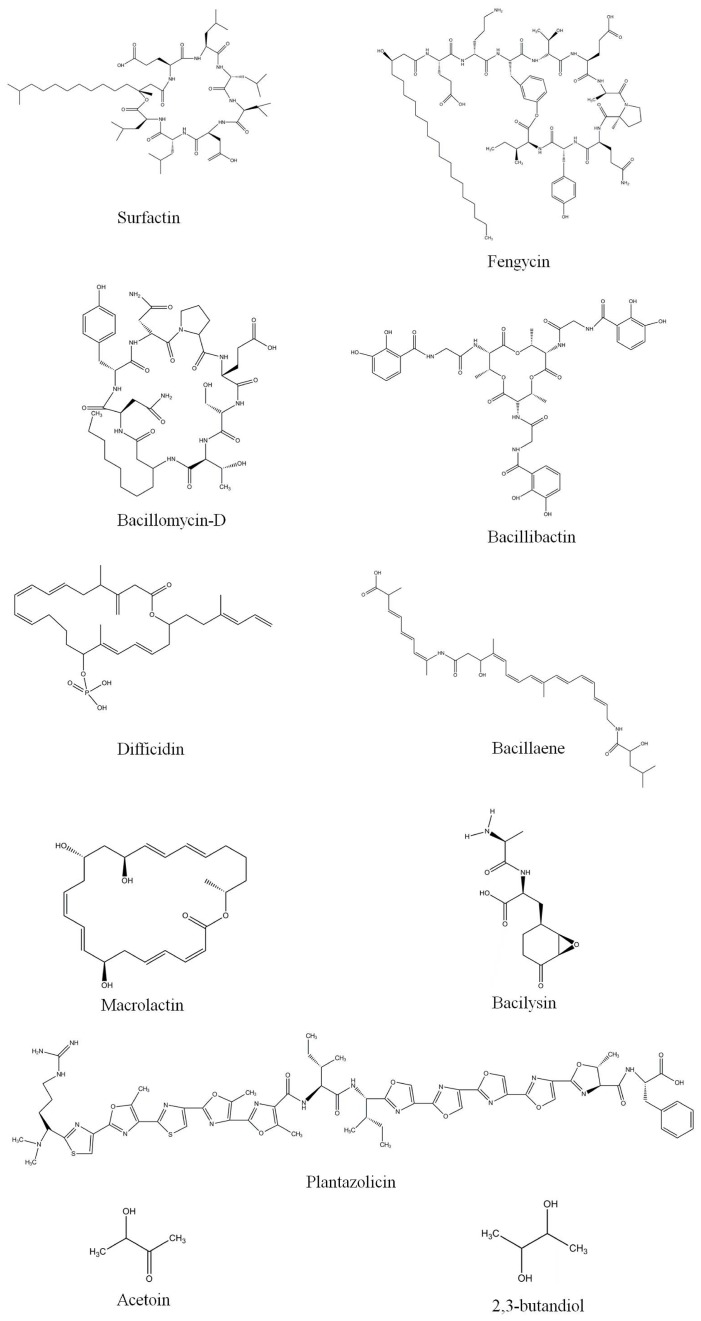
Molecular structure of ribosomal and nonribosomal bioactive compounds synthesized by *B. velezensis*.

In 2007, *B. amyloliquefaciens* FZB42 was reported as the first gram-positive biocontrol bacteria to have its genome sequenced [15]. The strain harbors an array of nine giant gene clusters that function to produce a spectrum of bioactive secondary metabolites (Figure 3) by modularly organized mega-enzymes, known as nonribosomal peptide synthetases and polyketide synthases (Table 1). Five of these nine gene clusters (i.e., *srf*, *bmy*, *fen*, *nrs*, and *dhb*; 137 kb) are involved in synthesizing cyclic lipopeptides molecules, such as surfactin, bacillomycin-D, fengycin, an unknown peptide, and the iron-siderophore bacillibactin. Meanwhile, three other gene clusters (i.e., *mln*, *bae*, and *dfn*; 199 kb) were reported to direct the synthesis of antibacterial polyketides, such as macrolactin, bacillaene, and difficidin, and the last gene cluster (*bac*; 6.9 kb) was reported to direct the synthesis and export of the antibacterial dipeptide bacilysin [29]. Altogether, about 10% (340 kb) of the *B. amyloliquefaciens* FZB42 genome is dedicated to the nonribosomal synthesis of lipopeptide and polyketide-type antimicrobial molecules, siderophores, and bacteriocins [29]. Closely related *Bacillus* species are also capable of synthesizing bioactive metabolites that exhibit activity against a wide range of microorganisms (Table 2).

### 3.1. Antibacterial Molecules

Difficidin and bacilysin are the most effective antibacterial agents produced by *B. amyloliquefaciens* FZB42 [7]. Little is known about the antibacterial properties of macrolactin and bacillaene [30]. *B. amyloliquefaciens* FZB42 exerts biocontrol activity by synthesizing difficidin and bacilysin, which facilitates the control of several economically important rice diseases, such as bacterial blight and bacterial leaf streak, which are caused by *Xanthomonas oryzae* pv. *oryzae* and *X. oryzae* pv. *oryzicola*, respectively [7]. *Erwinia amylovora*, the causative agent of fire blight disease, can also be effectively controlled by *B. amyloliquefaciens* FZB42 [31]. However, a mutant strain of *B. amyloliquefaciens* FZB42 that produces difficidin, but not macrolactin or bacillaene, was reported to exhibit equal or slightly higher activity against *E*. *amylovora* than the wild-type FZB42 strain. Bacilysin, one of the antibacterial molecules produced by *B. amyloliquefaciens* FZB42, is reported to exert an inhibitory effect against *E. amylovora* [31]. Bacilysin, also possesses anticyanobacterial activity against the harmful alga *Microcystis aeruginosa*, with a killing rate of 98.78%, and can, therefore, be used as a targeted biocontrol agent [32]. Meanwhile, bacillomycin-D and fengycin from *B. velezensis* might play redundant roles in defense mechanisms against *Ralstonia solanacearum*, an etiological agent of tomato wilting [33]. The expression of lipopeptide biosynthesis genes (*srfAB, ituC,* and *fenD* for the synthesis of surfactin, iturin, and fengycin, respectively) was greatly induced in co-cultures of *B. velezensis* and pathogens, such as *R. solanacearum* [33].

Bacteriocins are ribosomally synthesized peptidic toxins that are synthesized by bacteria. In the plant rhizosphere, such toxins may be produced to kill neighboring pathogenic microbes and nematodes, usually in response to environmental stresses [34,35]. The first known bacteriocin, colicin, was isolated from *Escherichia coli* by Gratia in 1925 [36]. Recent genomic analysis of *B*. *amyloliquefaciens* FZB42 revealed ribosomally encoded gene clusters for plantazolicin, a novel antibacterial and nematicidal agent [37], and amylocyclicin, an antibiotic (Table 1) [38]. Plantazolicin is synthesized by a cluster of 12 genes, which span nearly 10 kb of the *B*. *amyloliquefaciens* FZB42 genome [37], and amylocyclicin is a highly hydrophobic cyclic peptide that is synthesized by a cluster of six genes, which span nearly 4.5 kb, and are involved in the compound’s production, modification, exportation, and self-immunization [38].

### 3.2. Antifungal Molecules

The inoculation of lettuce seeds with commercial *B*. *amyloliquefaciens* FZB42 can reduce the severity of bottom rot disease caused by *Rhizoctonia solani* [20]. Ultra-performance liquid chromatography coupled with mass spectrometry suggested that this suppression might be due to the presence of cyclic lipopeptide molecules (i.e., surfactin, bacillomycin-D, and fengycin) in the lettuce root rhizosphere [20].

*B. amyloliquefaciens* FZB42 has been reported to exhibit antagonistic interactions with *Fusarium graminearum,* a plant-pathogenic fungus that threatens the production and quality of wheat and barley worldwide [39]. The antifungal activity exerted by *B. amyloliquefaciens* FZB42 has primarily been attributed to the nonribosomal synthesis of lipopeptide compounds [40]. For example, a bacillomycin-D deficient mutant strain of *B. amyloliquefaciens* FZB42 exhibited severely impaired antifungal activities, thereby suggesting that bacillomycin-D contributes significantly to the antifungal action of *B. amyloliquefaciens* FZB42 [29]. A double mutant of *B. amyloliquefaciens* FZB42 that was deficient in both bacillomycin-D and fengycin (Δ*bmyA* Δ*fenA*) was heavily impaired in its ability to inhibit the growth of *F*. *oxysporum*, thereby indicating synergistic effects among such lipopeptides against target organisms [41]. Bacillomycin-D also induces morphological changes in the plasma membranes and cell walls of *F. graminearum* hyphae and conidia [39].

### 3.3. Nematocidal Molecules

Plant-parasitic nematodes cause serious damage to many commercially important crops throughout the world [42]. Rhizospheric microorganisms control parasitic nematodes by secreting a variety of metabolites, enzymes, and toxins that suppress nematode reproduction, hatching, and juvenile survival [43]. A variety of nematophagous microbes, including *Bacillus* spp.*,* have been reported to possess nematicidal activity [44,45]. For example, treating tomato seedlings with *B. amyloliquefaciens* FZB42 reduced numbers of nematode eggs in tomato roots and of juvenile worms in soil and suppressed the incidence of tomato plant galls [44]. The nematicidal effect exerted by strain FZB42 has been attributed to plantazolicin, a novel compound encoded by the *pzn* gene cluster [45]. Culture filtrates of *B. amyloliquefaciens* FR203A have also been used as biocontrol agents for control of the nematode *Xiphinema index,* a pest of grape crops in Chile [24].

### 3.4. Siderophore Production

Iron is essential for growth in all living organisms, and most organisms depend on iron as a cofactor for important biochemical processes, including oxygen binding, electron transport, and catalysis [46]. In *B. amyloliquefaciens* FZB42, nonribosomal peptide synthetases are involved in the synthesis of siderophore bacillibactin [15], which play an important role in facilitating the acquisition of ferric ions (Fe^3+^) from minerals and organic compounds in the rhizosphere [46]. The binding of siderophores with environmentally free ferric ion facilitates the formation of siderophore–iron complexes that are transported back into bacterial cells through specific receptors (i.e., siderophore binding proteins) in the cell membrane. In gram-positive bacteria, siderophore-binding proteins, siderophore-permeases, and ATPases are involved in the transport of siderophore–iron complexes into the cytoplasm [46], where the ferric ions are reduced to ferrous (Fe^2+^) ions, thereby becoming available for microbial growth [47]. Rhizospheric *B. amyloliquefaciens* FZB42, which produces high concentrations of the siderophore bacillibactin, inhibits the growth of phytopathogenic bacterial and fungal competitors by depriving them of essential iron ions [15].

### 3.5. Production of Volatile Organic Compounds (VOCs)

VOCs are a complex mixture of low-molecular-weight, odorous, lipophilic compounds that are usually produced by plants and microorganisms [48]. Plant- and soil-associated microorganisms that produce VOCs have been reported to suppress virulent microbes, thereby indicating their potential as biocontrol agents against plant diseases [49]. Indeed, the VOCs released by *B. amyloliquefaciens* FZB42 possess antimicrobial activity and also promote plant growth and systemic resistance [50], whereas the VOCs, acetoin, and 2,3- butanediol, in particular, released by *B. subtilis* GB03 stimulate ISR in *Arabidopsis* seedlings and reduce the severity of disease in seedlings challenged with the soft rot pathogen *E. carotovora* subsp. *carotovora* [28,51].

The VOCs produced by *B. amyloliquefaciens* NJN-6 also inhibit fungal growth by suppressing mycelial growth and spore germination in *F. oxysporum* [26]. In fact, *F*. *oxysporum* growth was inhibited by approximately 30–40% [26]. In a similar study, a combination of 22 VOCs produced by *B. amyloliquefaciens* SQR9 were found to inhibit the growth of the tomato wilt pathogen *R. solanacearum* by 70% [52], and proteomic analysis indicated that the VOCs also affected pathogen virulence by downregulating catalase and superoxide dismutase activities. The SQR9 VOCs also reduced motility, biofilm formation, tomato root colonization by *R. solanacearum*, thereby clearly demonstrating the importance of VOCs in controlling pathogenic microbes [52].

## 4. Stimulation of Induced Systemic Resistance (ISR) by *B. velezensis*

**Figure 4:**
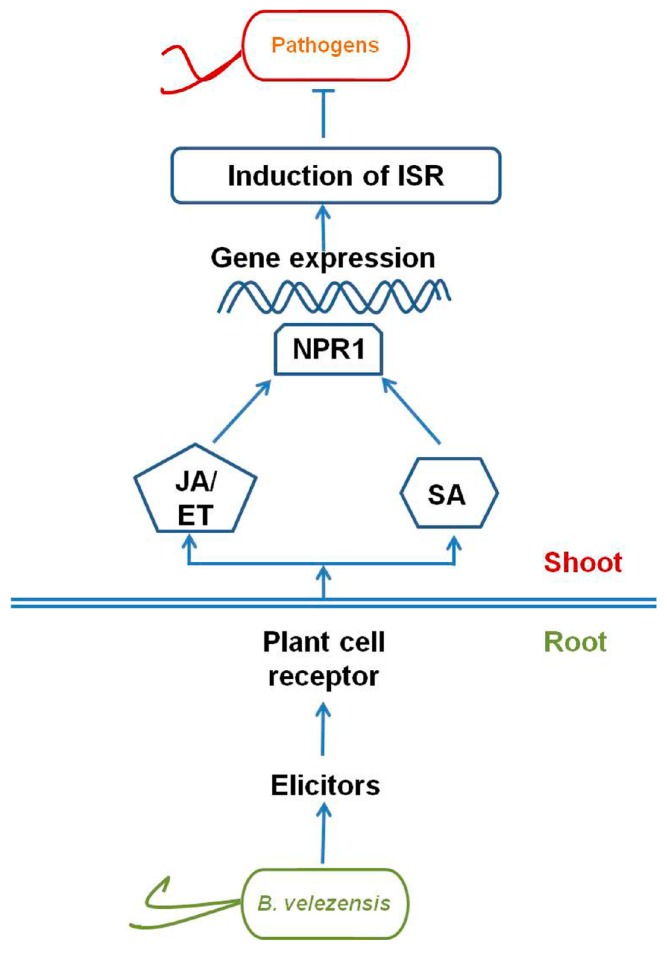
Signal transduction pathway of induced systemic resistance stimulated by *B. velezensis*. NPR1: non-expressor of PR1; JA/ET: the jasmonic acid/ethylene signaling pathways; SA: Salicylic Acid.

The application of PGPR to seeds or seedlings can stimulate ISR in the treated plants upon recognition of pathogens (Figure 4). The term ISR was first coined by van Peer et al. [62] to describe the resistance of carnation plants, in which the root had been previously treated with the rhizobacterium *Pseudomonas* WCS417r, against *F. oxysporum* f. sp. *dianthi*. The process of ISR depends on the recognition of PGPR-secreted elicitors, such as lipopolysaccharides, peptidoglycans, flagellin, quorum-sensing molecules, cyclic lipopeptides, and iron chelating siderophores [63,64]. 

It is now recognized that PGPR induce ISR in plants through the jasmonic acid/ethylene (JA/ET) signaling pathways [5,65] (Figure 4) and that the cellular defense responses of plants include oxidative bursts, cell-wall reinforcement, the accumulation of defense-related enzymes, and the production of antimicrobial phytoalexins [66]. The well-characterized JA/ET responsive genes of *Arabidopsis* include *LOX* (lipoxygenase), *VSP* (vegetative storage protein)*, PDF1.2* (plant defensin factor 1.2)*, Hel* (hevein), *CHI* (chitinase), and *PAL* (phenylalanine ammonia lyase) [67]. Interactions between PGPR and host plants can also activate both the JA/ET and salicylic acid signaling pathways, which are intertwined molecularly through their reliance on a functional version of *NPR1* (non-expressor of PR1), a gene that encodes a defense-related regulatory protein (Figure 4). For example, the PGPR *B. cereus* AR156 induces the simultaneous expression of the JA/ET-responsive marker gene *PDF1.2* and the salicylic acid-responsive marker genes *PR1*, *PR2*, and *PR5* in the leaves of *Arabidopsis thaliana* when exposed to the pathogen *P. syringae* pv. *tomato* DC3000 [68]. 

Surfactin and other nonribosomally synthesized secondary metabolites that are produced by *B. amyloliquefaciens* FZB42 have been reported to enhance plant defense responses in the root rhizosphere [20]. To examine the role of surfactin in the regulation of plant defense responses against *R. solani*, lettuce seedlings were bacterized with *B*. *amyloliquefaciens* FZB42 and two mutant strains of *B. amyloliquefaciens* FZB42, namely, CH1 (surfactin deficient) and CH5 (lipopeptide and polyketide deficient). Quantitative real-time PCR analysis indicated that *PDF1.2* was upregulated in the FZB42-bacterized plants in presence of *R. solani* but not in plants bacterized with the mutant strains [20].

In another study, a soil drench that contained both *B. amyloliquefaciens* SQRT3 and the pathogen *R. solanacearum* upregulated the expression of several defense-related marker genes (i.e., *Pin2*, *PR-1a,* and *Osmotin-like*) in tomato leaves over that observed in the control plants (drenched with *R. solanacearum* only). *B. amyloliquefaciens* SQRT3-mediated ISR was also reported to involve JA, salicylic acid, and ET-dependent signaling pathways and to reduce tomato bacterial wilt by 68.1% [27].

## 5. Biofilm Formation by *B. velezensis*

In the rhizosphere, plants create environments that are nutritionally and physicochemically beneficial for root microflora by continuously releasing a variety of organic molecules. Some of these molecules function as chemical signals that attract motile bacteria to move towards the root surface (i.e., chemotaxis), thereby favoring the formation of biofilms [5], which are aggregations of cells that live on either liquid or solid surfaces in a sticky, self-produced matrix of hydrated extracellular polymeric substances. Such extracellular polymeric substances are composed of polysaccharides, proteins*,* nucleic acids, and lipids and mainly function to facilitate cell stability, adhesion, cohesion, interconnection, and transient immobilization of cells in a biofilm [69].

The formation of biofilms in plant rhizospheres can promote plant growth and protect plants from infectious microbes, both through the secretion of antimicrobial compounds and through systemic resistance. For example, when *A. thaliana* is infected with *P. syringae*, the plant secretes malic acid in order to recruit rhizospheric bacteria and, consequently, to enhance *Bacillus* biofilm formation, thereby promoting immunity against phytopathogenic microbes [70]. The formation of biofilm by *B. amyloliquefaciens* SQR9 in liquid culture could be regulated by maize root exudates that contained glucose, citric acid, and fumaric acid, and transcriptional profiling of the SQR9 strain revealed that the maize root exudates activated the expression of genes related to extracellular matrix production. Several genes related to fengycin, bacillibactin, and bacilysin synthesis were also upregulated as a result of biofilm formation, thereby providing further evidence of the beneficial role of SQR9 in the maize rhizosphere [25]. As the global transcription regulator, AbrB has been shown to negatively regulate chemotaxis and biofilm formation in *Bacillus* [25], and the disruption of *abrB* in *B. amyloliquefaciens* SQR9 was reported to significantly increase biofilm formation and biocontrol ability [71]. When compared with control seedlings, symptoms of *Fusarium* wilt were reduced to 50% in *B. amyloliquefaciens* SQR9-treated cucumber seedlings, and to as low as 20% in seedlings that had been treated with the *abrB* mutant of *B. amyloliquefaciens* SQR9 [71].

These results suggest that *B. velezensis* can also reduce the severity of plant diseases by forming biofilms and that the disease-controlling capacity of the strain can be improved by deleting genes that negatively regulate chemotaxis and biofilm formation.

## 6. Conclusions and Future Prospects

Increased use of chemical fertilizers and pesticides has resulted in the accumulation of residual chemical compounds in the environment, and pathogenic microorganisms are starting to develop resistance. To circumvent these undesirable effects, it is of utmost importance to use biological agents, such as bio-fertilizers and bio-pesticides. Among the closely related *Bacillus* species, *B. velezensis* is attracting attention as a valuable biocontrol agent. Accordingly, in order to develop and formulate bio-based products, it is increasingly important to understand the antimicrobial potential of biosynthesis of *B*. *velezensis*. Furthermore, the elucidation of genes responsible for bioactive secondary metabolites and the ability to control such genes are additional important steps for increasing the production of metabolites by beneficial microbes and for facilitating metabolic engineering. *B. velezensis* may represent a practical and powerful biocontrol agent that can be used as an effective alternative to synthetic agro-chemicals.

## Figures and Tables

**Table 1:** Genes and gene clusters encoding for the secondary metabolites in *B. velezensis.*

Metabolite	Genes and gene clusters	Enzyme	Size (kb)	Functions	Controlling Effects	References
Nonribosomal synthesis of lipopeptides (LP)						
Surfactin	*srfABCD*	NRPS	32.0	Biofilm, Induction of ISR	Fungi	[20]
Fengycin	*fenABCDE*	NRPS	38.2	Induction of ISR	Fungi	[41]
Bacillomycin-D	*bmyCBAD*	NRPS/PKS	39.7	Induction of ISR	Fungi	[33,41]
Bacillibactin	*dhbABCDEF*	NRPS	12.8	Siderophore production	Microbial competitors	[53]
Nonribosomal synthesis of polyketides (PK)						
Difficidin	*dfnAYXBCDEFGHIJKLM*	NRPS	71.1	Direct suppression	Bacteria	[7,40]
Bacillaene	*baeBCDE*, *acpK*, *baeGHIJLMNRS*	PKS/NRPS	74.3	Direct suppression	Bacteria	[40]
Macrolactin	*mlnABCDEFGHI*	NRPS	53.9	Direct suppression	Bacteria	[30,54]
Nonribosomal synthesis of dipeptide antibiotics						
Bacilysin	*bacABCDE, ywfG*	NRPS	6.9	Direct suppression	Bacteria, Cyanobacteria	[55]
Ribosomal synthesis of bacteriocins						
Plantazolicin	*pznFKGHIAJC DBEL*	-	9.96	Direct suppression	Bacteria, Nematodes	[37,56]
Amylocyclicin	*ac* *n* *BACDEF*	-	4.49	Direct suppression	Bacteria	[38]
Synthesis of VOCs						
Acetoin and 2,3-butandiol	*alsSD; bdhA*	Acetolactate synthase/decarboxylase and 2,3-butanediol dehydrogenase	3.6	Induction of ISR	Bacteria, Fungi	[28,52]

NRPS = nonribosomal peptide synthetases; PKS = polyketide synthases; ISR = induced systemic resistance; VOCs: volatile organic compounds.

**Table 2:** List of various bioactive metabolites synthesized by *B. velezensis* and the closely related species.

*Bacillus* Species	Bioactive Metabolites	References
***B. velezensis***	Amylocyclicin, Bacilysin, Bacillomycin-D, Bacillibactin, Bacillaene, Difficidin, Fengycin, Macrolactin, Plantazolicin, Surfactin	[15]
***B. subtilis***	Bacillibactin, Bacillaene, Bacilysin, Difficidin, Bacitracin, Fengycin, Locillomycin, Subtilosin, Surfactin	[23,57]
***B. amyloliquefaciens*** **subsp.** ***amyloliquefaciens***	Bacillibactin, Bacillaene, Bacillomycin-D, Bacilysin, Fengycin, Surfactin	[23,58]
***B. siamensis***	Bacillomycin-D, Bacillaene, Difficidin, Fengycin, Surfactin	[59]
***B. licheniformis***	Bacitracin, Lichenysin, Lichenin	[60]
***B. pumilus***	Amicoumacin, Bacilysin, Bacircine, Pumilacidin	[61]

## References

[1] Sasse J., Martinoia E., Northen T. (2018). Feed your friends: Do plant exudates shape the root microbiome?. Trends Plant Sci..

[2] Hartmann A., Rothballer M., Schmid M. (2008). Lorenz Hiltner, a pioneer in rhizosphere microbial ecology and soil bacteriology research. Plant Soil.

[3] Morgan J.A.W., Bending G.D., White P.J. (2005). Biological costs and benefits to plant-microbe interactions in the rhizosphere. J. Exp. Bot..

[4] Beneduzi A., Ambrosini A., Passaglia L.M.P. (2012). Plant growth-promoting rhizobacteria (PGPR): Their potential as antagonists and biocontrol agents. Genet. Mol. Biol..

[5] Ongena M., Jacques P. (2008). *Bacillus* lipopeptides: Versatile weapons for plant disease biocontrol. Trends Microbiol..

[6] Chowdhury S.P., Dietel K., Rändler M., Schmid M., Junge H., Borriss R., Hartmann A., Grosch R. (2013). Effects of *Bacillus amyloliquefaciens* FZB42 on Lettuce growth and health under pathogen pressure and its impact on the rhizosphere bacterial community. PLoS ONE.

[7] Wu L., Wu H.J., Qiao J., Gao X., Borriss R. (2015). Novel routes for improving biocontrol activity of *Bacillus* based bioinoculants. Front. Microbiol..

[8] Pérez-García A., Romero D., de Vicente A. (2011). Plant protection and growth stimulation by microorganisms: Biotechnological applications of Bacilli in agriculture. Curr. Opin. Biotechnol..

[9] Cawoy H., Bettiol W., Fickers P., Ongena M. (2009). *Bacillus*-based biological control of plant diseases. Pestic. Mord. World-Pestic. Use Manag..

[10] Krebs B., Höding B., Kübart S., Workie M.A., Junge H., Schmiedeknecht G., Grosch R., Bochow H., Hevesi M. (1998). Use of *Bacillus subtilis* as biocontrol agent. I. Activities and characterization of *Bacillus subtilis* strains. Zeitschrift fur Pflanzenkrankheiten und Pflanzenschutz.

[11] Romanazzi G., Feliziani E., Bautista-Banos S. (2014). *Botrytis cinerea* (*Gray Mold*). Postharvest Decay: Control Strategies.

[12] Gordon R.E., Haynes W.C., Pang C.H.-N. (1973). The genus Bacillus.

[13] Priest F.G., Goodfellow M., Shute L.A., Berkeley R.C.W. (1987). *Bacillus amyloliquefaciens* sp. nov., nom. rev.. Int. J. Syst. Bacteriol..

[14] Fritze D. (2004). Taxonomy of the genus *Bacillus* and related genera: The aerobic endospore-forming bacteria. Phytopathology.

[15] Chen X.H., Koumoutsi A., Scholz R., Eisenreich A., Schneider K., Heinemeyer I., Morgenstern B., Voss B., Hess W.R., Reva O. (2007). Comparative analysis of the complete genome sequence of the plant growth-promoting bacterium *Bacillus amyloliquefaciens* FZB42. Nat. Biotechnol..

[16] Ruiz-García C., Béjar V., Martínez-Checa F., Llamas I., Quesada E. (2005). *Bacillus velezensis* sp. nov., a surfactant-producing bacterium isolated from the river Vélez in Málaga, southern Spain. Int. J. Syst. Evol. Microbiol..

[17] Sumpavapol P., Tongyonk L., Tanasupawat S., Chokesajjawatee N., Luxananil P., Visessanguan W. (2010). *Bacillus siamensis* sp. nov., isolated from salted crab (*poo-khem*) in Thailand. Int. J. Syst. Evol. Microbiol..

[18] Madhaiyan M., Poonguzhali S., Kwon S.W., Sa T.M. (2010). *Bacillus methylotrophicus* sp. nov., a methanol-utilizing, plant-growth-promoting bacterium isolated from rice rhizosphere soil. Int. J. Syst. Evol. Microbiol..

[19] Borriss R., Chen X.H., Rueckert C., Blom J., Becker A., Baumgarth B., Fan B., Pukall R., Schumann P., Sproer C. (2011). Relationship of *Bacillus amyloliquefaciens* clades associated with strains DSM 7^T^ and FZB42^T^: A proposal for *Bacillus amyloliquefaciens* subsp. *amyloliquefaciens* subsp. nov. and *Bacillus amyloliquefaciens* subsp. *plantarum* subsp. nov. based on complete genome sequence comparisons. Int. J. Syst. Evol. Microbiol..

[20] Chowdhury S.P., Uhl J., Grosch R., Alquéres S., Pittroff S., Dietel K., Schmitt-Kopplin P., Borriss R., Hartmann A. (2015). Cyclic lipopeptides of *Bacillus amyloliquefaciens* subsp. *plantarum* colonizing the Lettuce rhizosphere enhance plant defense responses toward the bottom rot pathogen *Rhizoctonia solani*. Mol. Plant-Microbe Interact..

[21] Dunlap C.A., Kim S.J., Kwon S.W., Rooney A.P. (2015). Phylogenomic analysis shows that *Bacillus amyloliquefaciens* subsp. *plantarum* is a later heterotypic synonym of *Bacillus methylotrophicus*. Int. J. Syst. Evol. Microbiol..

[22] Dunlap C.A., Kim S.J., Kwon S.W., Rooney A.P. (2016). *Bacillus velezensis* is not a later heterotypic synonym of *Bacillus amyloliquefaciens*; *Bacillus methylotrophicus*, *Bacillus amyloliquefaciens* subsp. *plantarum* and ‘*Bacillus oryzicola*’ are later heterotypic synonyms of *Bacillus velezensis* based on phylogenomics. Int. J. Syst. Evol. Microbiol..

[23] Fan B., Blom J., Klenk H.P., Borriss R. (2017). *Bacillus amyloliquefaciens*, *Bacillus velezensis*, and *Bacillus siamensis* form an “operational group *B. amyloliquefaciens*” within the *B. subtilis* species complex. Front. Microbiol..

[24] Castaneda-Alvarez C., Prodan S., Rosales I.M., Aballay E. (2016). Exoenzymes and metabolites related to the nematicidal effect of rhizobacteria on *Xiphinema index* Thorne & Allen. J. Appl. Microbiol..

[25] Zhang N., Yang D., Wang D., Miao Y., Shao J., Zhou X., Xu Z., Li Q., Feng H., Li S. (2015). Whole transcriptomic analysis of the plant-beneficial rhizobacterium *Bacillus amyloliquefaciens* SQR9 during enhanced biofilm formation regulated by maize root exudates. BMC Genom..

[26] Yuan J., Raza W., Shen Q., Huang Q. (2012). Antifungal activity of *Bacillus amyloliquefaciens* NJN-6 volatile compounds against *Fusarium oxysporum* f. sp. *cubense*. Appl. Environ. Microbiol..

[27] Li C.Y., Hu W.C., Pan B., Liu Y., Yuan S.F., Ding Y.Y., Li R., Zheng X.Y., Shen Q.R. (2017). Rhizobacterium *Bacillus amyloliquefaciens* strain SQRT3-mediated induced systemic resistance controls bacterial wilt of Tomato. Pedosphere.

[28] Ryu C.-M., Farag M.A., Hu C.-H., Reddy M.S., Kloepper J.W., Pare P.W. (2004). Bacterial volatiles induce systemic resistance in *Arabidopsis*. Plant Physiol..

[29] Chen X.H., Koumoutsi A., Scholz R., Schneider K., Vater J., Süssmuth R., Piel J., Borriss R. (2009). Genome analysis of *Bacillus amyloliquefaciens* FZB42 reveals its potential for biocontrol of plant pathogens. J. Biotechnol..

[30] Romero-Tabarez M., Jansen R., Sylla M., Lünsdorf H., Häussler S., Santosa D.A., Timmis K.N., Molinari G. (2006). 7-*O*-malonyl macrolactin A, a new macrolactin antibiotic from *Bacillus subtilis* active against methicillin-resistant *Staphylococcus aureus*, vancomycin-resistant enterococci, and a small-colony variant of *Burkholderia cepacia*. Antimicrob. Agents Chemother..

[31] Chen X.H., Scholz R., Borriss M., Junge H., Mögel G., Kunz S., Borriss R. (2009). Difficidin and bacilysin produced by plant-associated *Bacillus amyloliquefaciens* are efficient in controlling fire blight disease. J. Biotechnol..

[32] Wu L., Wu H., Chen L., Xie S., Zang H., Borriss R., Gao X. (2014). Bacilysin from *Bacillus amyloliquefaciens* FZB42 has specific bactericidal activity against harmful algal bloom species. Appl. Environ. Microbiol..

[33] Cao Y., Pi H., Chandrangsu P., Li Y., Wang Y., Zhou H., Xiong H., Helmann J.D., Cai Y. (2018). Antagonism of two plant-growth promoting *Bacillus velezensis* isolates against *Ralstonia solanacearum* and *Fusarium oxysporum*. Sci. Rep..

[34] White P., Joshi A., Rassam P., Housden N.G., Kaminska R., Goult J.D., Redfield C., McCaughey L.C., Walker D., Mohammed S. (2017). Exploitation of an iron transporter for bacterial protein antibiotic import. Proc. Natl. Acad. Sci. USA.

[35] Arias A.A., Ongena M., Devreese B., Terrak M., Joris B., Fickers P. (2013). Characterization of amylolysin, a novel lantibiotic from *Bacillus amyloliquefaciens* GA1. PLoS ONE.

[36] Cascales E., Buchanan S.K., Duche D., Kleanthous C., Lloubes R., Postle K., Riley M., Slatin S., Cavard D. (2007). Colicin Biology. Microbiol. Mol. Biol. Rev..

[37] Scholz R., Molohon K.J., Nachtigall J., Vater J., Markley A.L., Süssmuth R.D., Mitchell D.A., Borriss R. (2011). Plantazolicin, a novel microcin B17/streptolysin S-like natural product from *Bacillus amyloliquefaciens* FZB42. J. Bacteriol..

[38] Scholz R., Vater J., Budiharjo A., Wang Z., He Y., Dietel K., Schwecke T., Herfort S., Lasch P., Borriss R. (2014). Amylocyclicin, a novel circular bacteriocin produced by *Bacillus amyloliquefaciens* FZB42. J. Bacteriol..

[39] Gu Q., Yang Y., Yuan Q., Shi G., Wu L., Lou Z., Huo R., Wu H., Borriss R., Gao X. (2017). Bacillomycin D produced by *Bacillus amyloliquefaciens* is involved in the antagonistic interaction with the plant-pathogenic fungus *Fusarium graminearum*. Appl. Environ. Microbiol..

[40] Chen X.H., Vater J., Piel J., Franke P., Scholz R., Schneider K., Koumoutsi A., Hitzeroth G., Grammel N., Strittmatter A.W. (2006). Structural and functional characterization of three polyketide synthase gene clusters in *Bacillus amyloliquefaciens* FZB42. J. Bacteriol..

[41] Koumoutsi A., Chen X.H., Henne A., Liesegang H., Hitzeroth G., Franke P., Vater J., Borriss R. (2004). Structural and functional characterization of gene clusters directing nonribosomal synthesis of bioactive cyclic lipopeptides in *Bacillus amyloliquefaciens* strain FZB42. J. Bacteriol..

[42] Koenning S.R., Overstreet C., Noling J.W., Donald P.A., Becker J.O., Fortnum B.A. (1999). Survey of crop losses in response to phytoparasitic nematodes in the United States for 1994. J. Nematol..

[43] Tian B., Yang J., Zhang K.Q. (2007). Bacteria used in the biological control of plant-parasitic nematodes: Populations, mechanisms of action, and future prospects. FEMS Microbiol. Ecol..

[44] Burkett-Cadena M., Kokalis-Burelle N., Lawrence K.S., van Santen E., Kloepper J.W. (2008). Suppressiveness of root-knot nematodes mediated by rhizobacteria. Biol. Control.

[45] Liu Z., Budiharjo A., Wang P., Shi H., Fang J., Borriss R., Zhang K., Huang X. (2013). The highly modified microcin peptide plantazolicin is associated with nematicidal activity of *Bacillus amyloliquefaciens* FZB42. Appl. Microbiol. Biotechnol..

[46] Fukushima T., Allred B.E., Sia A.K., Nichiporuk R., Andersen U.N., Raymond K.N. (2013). Gram-positive siderophore-shuttle with iron-exchange from Fe-siderophore to apo-siderophore by *Bacillus cereus* YxeB. Proc. Natl. Acad. Sci. USA.

[47] Saha R., Saha N., Donofrio R.S., Bestervelt L.L. (2013). Microbial siderophores: A mini review. J. Basic Microbiol..

[48] Schulz-Bohm K., Martín-Sánchez L., Garbeva P. (2017). Microbial volatiles: Small molecules with an important role in intra- and inter-kingdom interactions. Front. Microbiol..

[49] Ossowicki A., Jafra S., Garbeva P. (2017). The antimicrobial volatile power of the rhizospheric isolate *Pseudomonas donghuensis* P482. PLoS ONE.

[50] Compant S., Duffy B., Nowak J., Cle´ment C., Barka E.A. (2005). Use of plant growth-promoting bacteria for biocontrol of plant diseases: Principles, mechanisms of action, and future prospects. Appl. Environ. Microbiol..

[51] Ryu C.M., Farag M.A., Hu C.H., Reddy M.S., Wei H.X., Paré P.W., Kloepper J.W. (2003). Bacterial volatiles promote growth in *Arabidopsis*. Proc. Natl. Acad. Sci. USA.

[52] Raza W., Ling N., Yang L., Huang Q., Shen Q. (2016). Response of Tomato wilt pathogen *Ralstonia solanacearum* to the volatile organic compounds produced by a biocontrol strain *Bacillus amyloliquefaciens* SQR-9. Sci. Rep..

[53] Li Y., Gu Y., Li J., Xu M., Wei Q., Wang Y. (2015). Biocontrol agent *Bacillus amyloliquefaciens* LJ02 induces systemic resistance against Cucurbits powdery mildew. Front. Microbiol..

[54] Schneider K., Chen X.H., Vater J., Franke P., Nicholson G., Borriss R., Süssmuth R.D. (2007). Macrolactin is the polyketide biosynthesis product of the pks2 cluster of *Bacillus amyloliquefaciens* FZB42. J. Nat. Prod..

[55] Wu L., Wu H., Chen L., Yu X., Borriss R., Gao X. (2015). Difficidin and bacilysin from *Bacillus amyloliquefaciens* FZB42 have antibacterial activity against *Xanthomonas oryzae* rice pathogens. Sci. Rep..

[56] Kalyon B., Helaly S.E., Scholz R., Nachtigall J., Vater J., Borriss R., Süssmuth R.D. (2011). Plantazolicin A and B: Structure elucidation of ribosomally synthesized thiazole/oxazole peptides from *Bacillus amyloliquefaciens* FZB42. Org. Lett..

[57] Guo S., Li X., He P., Ho H., Wu Y., He Y. (2015). Whole-genome sequencing of *Bacillus subtilis* XF-1 reveals mechanisms for biological control and multiple beneficial properties in plants. J. Ind. Microbiol. Biotechnol..

[58] Rückert C., Blom J., Chen X.H., Reva O., Borriss R. (2011). Genome sequence of *B. amyloliquefaciens* type strain DSM7^T^ reveals differences to plant-associated *B. amyloliquefaciens* FZB42. J. Biotechnol..

[59] Jeong H., Jeong D.E., Kim S.H., Song G.C., Park S.Y., Ryu C.M., Park S.H., Choi S.K. (2012). Draft genome sequence of the plant growth-promoting bacterium *Bacillus siamensis* KCTC 13613T. J. Bacteriol..

[60] Harwood C.R., Mouillon J.-M., Pohl S., Arnau J. (2018). Secondary metabolite production and the safety of industrially important members of the *Bacillus subtilis* group. FEMS Microbiol. Rev..

[61] Sansinenea E., Ortiz A. (2011). Secondary metabolites of soil *Bacillus* spp.. Biotechnol. Lett..

[62] Van Peer R., Niemann G.J., Schippers B. (1991). Induced resistance and phytoalexin accumulation in biological control of fusarium wilt of Carnation by *Pseudomonas* sp. strain WCS417r. Phytopathology.

[63] Bakker P.A.H.M., Pieterse C.M.J., van Loon L.C. (2007). Induced systemic resistance by fluorescent *Pseudomonas* spp.. Phytopathology.

[64] Verhagen B., Trotel-Aziz P., Jeandet P., Baillieul F., Aziz A. (2011). Improved resistance against *Botrytis cinerea* by grapevine-associated bacteria that induce a prime oxidative burst and phytoalexin production. Phytopathology.

[65] Van Loon L.C. (2007). Plant responses to plant growth-promoting rhizobacteria. Eur. J. Plant Pathol..

[66] Park K., Park Y.-S., Ahamed J., Dutta S., Ryu H., Lee S.-H., Balaraju K., Manir M., Moon S.-S. (2016). Elicitation of induced systemic resistance of chili pepper by iturin A analogs derived from *Bacillus vallismortis* EXTN-1. Can. J. Plant Sci..

[67] Verhagen B.W.M., Glazebrook J., Zhu T., Chang H.-S., van Loon L.C., Pieterse C.M.J. (2004). The transcriptome of rhizobacteria-induced systemic resistance in *Arabidopsis*. Mol. Plant-Microbe Interact..

[68] Niu D.-D., Liu H.-X., Jiang C.-H., Wang Y.-P., Wang Q.-Y., Jin H.-L., Guo J.-H. (2011). The plant growth–promoting rhizobacterium *Bacillus cereus* AR156 induces systemic resistance in *Arabidopsis thaliana* by simultaneously activating salicylate -and jasmonate/ethylene-dependent signaling pathways. Mol. Plant-Microbe Interact..

[69] Bleich R., Watrous J.D., Dorrestein P.C., Bowers A.A., Shank E.A. (2015). Thiopeptide antibiotics stimulate biofilm formation in *Bacillus subtilis*. Proc. Natl. Acad. Sci. USA.

[70] Rudrappa T., Czymmek K.J., Pare P.W., Bais H.P. (2008). Root-secreted malic acid recruits beneficial soil bacteria. Plant Physiol..

[71] Weng J., Wang Y., Li J., Shen Q., Zhang R. (2013). Enhanced root colonization and biocontrol activity of *Bacillus amyloliquefaciens* SQR9 by *abrB* gene disruption. Appl. Microbiol. Biotechnol..

